# Evaluation of Tumor Budding in Primary Colorectal Cancer and Corresponding Liver Metastases Based on H&E and Pancytokeratin Staining

**DOI:** 10.3389/fmed.2019.00247

**Published:** 2019-10-31

**Authors:** Annika Blank, Carla Schenker, Heather Dawson, Guido Beldi, Inti Zlobec, Alessandro Lugli

**Affiliations:** ^1^Institute of Pathology, University of Bern, Bern, Switzerland; ^2^Department of Visceral Surgery and Medicine, Inselspital, Bern University Hospital, University of Bern, Bern, Switzerland

**Keywords:** tumor budding, metastasis, intratumoral budding, intrametastatic budding, peritumoral budding, perimetastatic budding

## Abstract

In colorectal cancer, tumor budding is associated with tumor progression and represents an additional prognostic factor in the TNM classification. Tumor buds can be found at the invasive front (peritumoral budding; PTB) and in the tumor center (intratumoral budding; ITB) of primary tumors. Previous studies have shown that tumor buds are also present in colorectal liver metastases (CRLM). Data on the prognostic and predictive role in this clinical context are still sparse and no standardized approach to evaluate budding in CRLM has been published so far. This study aimed to analyze and correlate perimetastatic (PMB) and intrametastatic budding (IMB) on H&E and pancytokeratin staining, compare it to budding results in corresponding primary tumors and to propose a standardized scoring system in CRLM as the basis for future studies. Tumor tissue of 81 primary tumors and 139 corresponding CRLM was used for ngTMA construction. For each primary tumor and metastasis, two punches from the center and two punches from the periphery from areas with highest tumor budding density were included. TMA slides were stained for H&E and pancytokeratin (Pan-CK). PTB, ITB, PMB, and IMB were analyzed and classified as bd1, bd2, and bd3 according to ITBCC guidelines. ITB and PTB as well as IMB and PMB showed significant correlation on H&E and Pan-CK staining. No correlation was found for tumor bud counts in primary tumors and corresponding metastases. The agreement for categorized tumor bud counts showed fair to good agreement for metastases and poor agreement for primary tumors between different classes on H&E and Pan-CK staining. Based on our results, tumor budding in primary tumors and CRLM seems to be different processes which might be the results of differing surrounding microenvironments. The evaluation of tumor budding in CRLM is challenging in cases without desmoplastic stroma reaction or intense perimetastatic ductular reaction. We therefore propose to evaluate tumor budding only in metastases with desmoplastic stroma reaction based on H&E staining since important morphological features are obscured on Pan-CK staining.

## Introduction

In colorectal cancer, tumor budding is associated with tumor progression, local and distant metastases (1) and is an additional prognostic factor in the TNM classification published by the UICC (2). In 2016, the international tumor budding consensus conference (ITBCC) proposed a standardized scoring system, validated by several studies over the last few years (3–12). Additionally, the ITBCC highlighted the importance of tumor budding especially in two clinical scenarios: in pT1 CRC, tumor budding may be an indicator of an oncologic resection and in stage II CRC of adjuvant therapy, respectively (13).

A geographic histological analysis of CRC revealed the presence of tumor buds not only at the invasive tumor front (peritumoral budding, PTB), but also within the main tumor body (intratumoral budding, ITB) (14). The clinical value of ITB is its potential assessment in preoperative rectal cancer biopsies (15) and the prognostic significance shown now by several studies (16, 17).

An additional clinical scenario for tumor budding may be the management of stage IV CRC. The treatment of colorectal cancer liver metastases (CRLM) includes surgery alone and/or a combination with systemic chemotherapy. In a recent study, tumor budding was analyzed on a monocentric patient cohort (*n* = 229) which underwent a first surgical resection of CRLM (18). Tumor budding was counted on H&E slides using a quantitative method selecting the area with highest density and counting sequential HPFs and shown to be a prognostic factor in univariate, but not in multivariate analysis (18). Nevertheless, there is not enough data in the literature to make final conclusions on the prognostic or predictive value of tumor budding in CRLM.

One of the main lessons learnt from the ITBCC is the stepwise validation of promising histological biomarkers and their potential value in daily practice. Therefore, we embarked in this preliminary study with three well-defined aims: first, to systematically analyze the geographic map of tumor budding in CRLM by introducing two terms, namely intrametastatic budding (IMB) and perimetastatic budding (PMB) and difficulties associated with the assessment of budding in hepatic resections; second, to score IMB and PMB on pan-cytokeratin (Pan-CK) and H&E stained slides based on the ITBCC method; third, to propose a scoring system for tumor budding in CRLM as a basis for future large multi-centric retrospective and prospective studies.

## Materials and Methods

### Patient Cohort

Histological slides from a retrospective cohort of initially 110 patients surgically treated between 2000 and 2016 at the Inselspital Bern for their primary CRC and synchronous or metachronous CRLM were screened for tumor budding. Tumors without tumor budding in either the primary CRC or corresponding CRLM were excluded from the cohort. The final cohort included 81 patients of which one patient had two metachronous primary CRC. Formalin-fixed paraffin-embedded tissues from 82 primary CRC and 139 corresponding CRLM were used for this study and their corresponding clinicopathological data are summarized in Table 1.

**Table 1:** Clinicopathological features.

**Clinicopathological features (*N* = 81)**
Gender
Male	55
Female	26
Histological subtype (primary)
Adeno	80
Mucinous	1
Tumor location (primary)
Left	44
Right	34
Rectum	1
Rectosigmoid	3
pT
pT1	0
pT2	6
pT3	52
pT4	23
pN
pN0	19
pN1-2	62
Tumor grade (primary)
G1-2	59
G3	18
Neoadjuvant therapy	4
Lymphatic invasion (primary)
L0	13
L1	39
Venous invasion (primary)
V0	19
V1	41
Perineural invasion (primary)
Pn0	24
Pn1	21
MMR status
Deficient	4
Proficient	77
Time to metastasis
Synchronous	56
Metachronous	25
Number of metastases
Median	2
Range	1–9

### Slide Scanning and Annotations

H&E slides of all cases were reviewed to identify tumor blocks from primary tumors and liver metastases with highest density of tumor buds at the tumor front and within the tumor. The tumor front was defined as the desmoplastic stroma surrounding the most advancing parts of the main tumor body. Only resection specimens were considered for the study.

Selected tumor blocks were re-cut and slides were stained for H&E. All H&E stained slides were scanned (Pannoramic P250, 3D Histech, Hungary, 20× objective lens) and uploaded onto a digital platform (http://ngtma.path.unibe.ch/casecenter). Digital slides were reviewed and areas with highest density of tumor budding were annotated using a TMA annotation tool (Panoramic viewer v15.1 and TMA annotation tool, 3D Histech, Hungary). Different colors for tumor front (blue color) and center (red color) were used. Two annotations from the tumor center and two annotations from the tumor front were placed onto the digital slides whenever possible.

### Next-Generation Tissue Microarray (ngTMA®) Construction

Eighty-two blocks from primary tumors and 144 blocks from liver metastases served as donor blocks for ngTMA construction. In CRLM with only few vital tumor cells it was necessary to include more than one tumor block. Donor blocks and annotated digital slides were loaded into an automated tissue microarrayer (Grandmaster, 3D Histech). An image of each donor block was taken and superimposed onto the digital slide for exact correspondence. After confirming each annotation, punches with a diameter of 1 mm from donor blocks were taken and transferred into a recipient block. A total of 328 punches from primary tumors (tumor front: 164; tumor center: 162) and a total number of 560 punches from liver metastases (tumor front: 284; tumor center: 276) were included in the ngTMA.

### Immunohistochemistry

TMA blocks were sectioned at 2.5 μm. Sections were mounted on glass slides, dried and baked at 60°C for 30 min. H&E staining and double immunohistochemistry for Pan-CK and CD8 were performed. Double immunohistochemistry was performed using Bond RX (Leica Biosystems). Slides were dewaxed using Bond dewax solution (product code AR9222, Leica Biosystems). Heat-induced epitope retrieval in citrate buffer based (code AR9640, Leica Biosystems) at pH6 for 20 min at 100°C was followed by incubation with primary mouse pancytokeratin antibody (Dako, clone AE1/AE3, Ref M351501-2); dilution 1:400; for 30 min. Slides were incubated with HRP (horseradish peroxidase)-polymer for 15 min. Visualization was accomplished using 3,3-Diaminobenzidine (DAB) for 10 min, leading to a brown staining signal (Bond polymer refine detection, Leica Biosystems, Ref DS9800). As a second step, mouse CD8 antibody was used (Dako-Agilent, clone C8/144B, Ref M7103); dilution 1:100; incubation time 30 min. Alkaline Phosphatase (AP)-polymer was used as secondary antibody; incubation time 15 min. Visualization was accomplished using fast red resulting in a red chromogen (Red polymer refine Detection, Leica Biosystems, Ref DS9390). Samples were counterstained with hematoxylin and mounted with Aquatex (Merck).

### Evaluation of H&E and Immunohistochemistry

**Figure 1:**
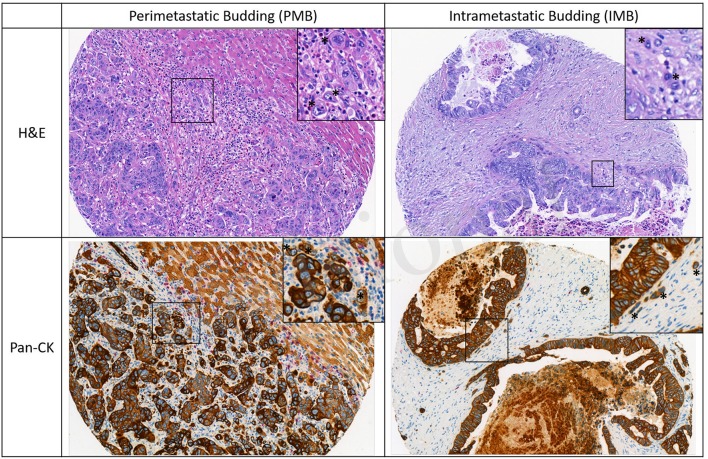
Representative images of perimetastatic and intrametastatic budding on H&E and Pan-CK staining. Examples of tumor buds are indicated with an asterisk.

All ngTMA slides were scanned (Pannoramic P250, 3DHistech, Hungary, 20× objective lens) and evaluated using Scorenado, a TMA analysis tool for digital TMA slides, as described previously (19). Each tumor punch contained an area of 0.785 mm^2^. Only Pan-CK staining was used to evaluated tumor budding in the present study. Tumor buds were defined as single cells or cell cluster of up to 4 tumor cells according to the ITBCC guidelines (13). One experienced pathologist (A.L.) evaluated the number of tumor buds on H&E and Pan-CK staining at the tumor front (PTB and PMB) as well as intratumoral (ITB and IMB). Representative examples for PMB and IMB are given in Figure 1.

### Statistical Analysis

Descriptive statistics were performed to determine the number of tumor buds in both H&E and CK, including mean, minimum and maximum values across the center and tumor front in both primary tumor and metastatic lesions. Pearson's correlation coefficient was used to determine the strength of linear association between budding counts. *P*-values <s 0.05 (two-sided) were considered statistically significant. Kappa statistics and 95%CI were used to investigate agreement in BD categories between primary and metastatic tumors. The agreement was again determined using intraclass correlation coefficients (ICC values) for raw budding counts. All analyses were performed using SAS v9.4, the SAS Institute (Cary, NC).

## Results

Tumor budding was assessed by counting all tumor buds per punch on H&E and Pan-CK staining. For each patient the mean, minimum and maximum number of ITB, PTB, IMB, and PMB was recorded. Results are summarized in Table 2. A significant difference was found between the mean number of tumor buds in primary tumors and metastases on Pan-CK staining but not on H&E (Table 3).

**Table 2:** Differences in the average number of ITB/IMB and PTB/PMB.

**Stain**	**Tissue**	**Center/Front**	**No**.	**Mean**	**Min**	**Max**
H&E	Primaries	Front	81	8.0	0	46
H&E	Primaries	Center	81	6.4	0	21
Pan-CK	Primaries	Front	81	12.3	0	53
Pan-CK	Primaries	Center	81	10.2	0	98
H&E	Metastases	Front	74	7.4	0	33
H&E	Metastases	Center	74	8.9	0	36
Pan-CK	Metastases	Front	74	9.0	0	59
Pan-CK	Metastases	Center	74	11.9	0	71

**Table 3:** Number of buds in total metastasis and primary with means and test of differences between matched samples.

	**Mean no. of buds**	***P*-value**
Primaries H&E	7.2	0.6229
Metastases H&E	8.3	
Primaries Pan-CK	11.3	0.0038
Metastases Pan-CK	7.3	

The correlation coefficients for ITB, PTB, IMB, and PMB on H&E and Pan-CK staining are included in Table 4. ITB and PTB showed significant correlation on H&E. IMB and PMB showed significant correlation on H&E and Pan-CK staining. No correlation was detected for tumor bud counts in primary tumors and corresponding metastases except for IMB on H&E staining in comparison to ITB on Pan-CK staining.

**Table 4:** Correlation of intratumoral and intrametastatic budding (ITB/IMB) with peritumoral and perimetastatic budding (PTB/PMB).

	**H&E PTB**	**H&E ITB**	**Pan-CK PTB**	**Pan-CK ITB**	**H&E PMB**	**H&E IMB**	**Pan-CK PMB**	**Pan-CK IMB**
H&E PTB	1.0							
H&E ITB	0.36^*^	1.0						
Pan-CK PTB	0.27^*^	0.11	1.0					
Pan-CK ITB	−0.12	0.04	0.36	1.0				
H&E PMB	0.03	0.11	−0.14	0.05	1.0			
H&E IMB	0.08	0.09	−0.06	0.25^*^	0.6^*^	1.0		
Pan-CK PMB	0.07	−0.08	−0.01	−0.01	0.44^*^	0.4^*^	1.0	
Pan-CK IMB	0.01	−0.03	0.01	0.07	0.3^*^	0.3^*^	0.44^*^	1.0

*Correlation coefficient indicated (r). Asterisk indicates p < 0.05*.

Tumor bud counts from primaries and metastases were categorized as bd1, bd2, and bd3 according to the ITBCC guidelines (13) on H&E as well as on Pan-CK. The agreement for categorized tumor bud counts were estimated using kappa values. For metastases there was fair to good agreement. For primary tumors, agreement was poor, between different classes on H&E and Pan-CK staining. Results are given in Table 5.

**Table 5:** Kappa values showing the concordance and percent agreement of bd scores for H&E and Pan-CK.

		**Pan-CK primaries front**			**Kappa (95% CI)**	**% Concordance**
		BD1	BD2	BD3		
**H&E primaries front**	BD1	12	3	14		
	BD2	4	4	13	0.14 (0–32)	42%
	BD3	4	9	18		
		**Pan-CK primaries center**			**Kappa (95% CI)**	**% Concordance**
		BD1	BD2	BD3		
**H&E primaries center**	BD1	13	8	13		
	BD2	8	2	13	0.03 (−0.2–0.14)	30%
	BD3	10	4	9		
		**Pan-CK metastases front**			**Kappa (95% CI)**	**% Concordance**
		BD1	BD2	BD3		
**H&E metastases front**	BD1	8	6	4	0.4 (0.2–0.6)	54%
	BD2	4	4	6		
	BD3	1	2	15		
		**Pan-CK metastases center**			**Kappa (95% CI)**	**% Concordance**
		BD1	BD2	BD3		
**H&E metastases center**	BD1	11	5	1	0.62 (0.4–0.8)	68.8%
	BD2	2	6	3		
	BD3	1	3	16		

## Discussion

The process of tumor budding in primary tumors and liver metastases seems to be different. Although there was an association between PTB and ITB as well as PMB and IMB, respectively, a correlation between tumor budding in the primary tumor and the corresponding metastases was not observed.

From a biological point of view, the present results could make sense based on the following hypothesis. The formation of tumor budding has been shown to be an important step in the process of epithelial mesenchymal transition (EMT) (20–22). As EMT is highly dependent on the tumor microenvironment, one could expect different pathogenetic mechanisms of budding in the liver parenchyma compared to the colorectal wall. The formation of buds might be advantageous in one organ, but obstructive or even destructive at the same time under different circumstances. Consequently, tumor budding in CRLM may differ from its role in primary CRC including the definition of cut offs for therapeutic decision making as well as a prognostic and predictive factor. The significant difference in the mean number of tumor buds between primary tumors and metastases on Pan-CK staining could provide further evidence for this hypothesis.

These assumptions are corroborated by differences in prognosis depending on the observed growth patterns in CRLM. Desmoplastic CRLM are associated with a better prognosis compared with the replacement or pushing type. The replacement type of CRLM demonstrates a close proximity to hepatic sinusoids and has been shown to be non-angiogenic. The desmoplastic and pushing type on the other hand reveal an angiogenic phenotype that might be disadvantageous in a highly vascularized organ like the liver (23–28). A broad rim of collagen could be even more hindering for tumor progression and thus represents an explanation for the observed differences in our study. Tumor cell migration and angiogenesis is an important factor for tumor progression in primary CRC but might be of no or lesser importance in CRLM.

Up to now there are two publications which demonstrate that tumor budding is a prognostic factor in CRLM based on univariate, but not in multi-variate analysis (18, 29). This result suggests that other factors such as differing components of the microenvironment, including inflammatory and stromal cells in the colon wall and in the liver may influence tumor budding and therefore have a more important prognostic and/or predictive role.

**Figure 2:**
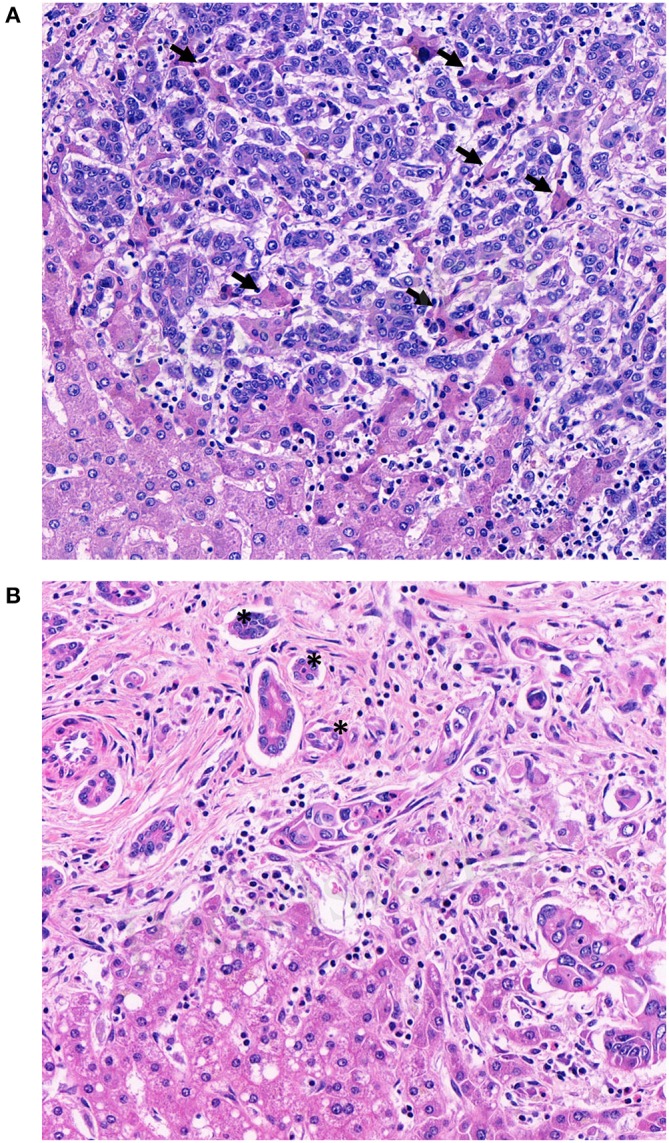
Methodical challenges for tumor budding evaluation in colorectal liver metastases. **(A)** Single cells or tumor cells clusters in between hepatocellular trabecula without obvious desmoplastic stroma reaction. Examples of hepatocellular trabecula between infiltrating tumor cells are indicated with arrows **(B)** Tumor buds and ductular reaction with reactive changes in close proximity. Examples of ductular reaction are indicated with an asterisk.

In primary CRC, pathologists using tumor budding in daily practice know potential pitfalls such as intense peritumoral inflammation, prominent stromal reaction with high numbers of activated macrophages and glandular fragmentation (30). Tumor buds are usually surrounded by desmoplastic stroma. If this is not the case, retraction artifacts or vascular invasion have to be ruled out. Pitfalls associated with the evaluation of tumor budding in CRLM have not been described, yet. Before using tumor budding in CRLM, its methodological challenges needs to be elucidated and discussed. The hepatic microscopic architecture differs significantly from the colon wall. The liver is a highly vascularized organ, consisting of numerous arteries, veins and sinusoids. These architectural differences permits growth patterns other than the ones known from primary tumors (25, 31, 32). Only the desmoplastic type of CRLM is likely to demonstrate tumor buds. The pushing type, by definition, should not manifest with single tumor cells or clusters at the invasive front. The replacement type can exhibit single cells or tumor cell clusters at the invasive front, but these are more likely to represent vascular invasion due to their non-angiogenic phenotype than actual tumor budding (Figure 2A). Second, CRLM are often surrounded by a prominent ductular reaction (Figure 2B) which can mimic tumor budding. Unfortunately, there is no reliable immunohistochemical marker to differentiate tumor buds from ductular reaction. Hence, the pathologist has to rely on morphological features like nuclear-cytoplasmic-ratio, anisokaryosis and hyperchromasia alone to distinguish bile ducts from tumor buds.

In summary, our main goal was to systematically analyze the tumor budding scoring systems in CRLM and therefore focus only on the methodological aspects and not on its predictive and prognostic role. Therefore, we suggest to evaluate tumor budding only in CRLM with desmoplastic stroma reaction on H&E stained slides using the ITBCC method because of two reasons: First, infiltrating tumor cells without surrounding stroma reaction cannot be reliably differentiated from vascular invasion. Second, morphological features to differentiate tumor buds from bile ducts are more easily detected by H&E than by immunohistochemistry. These aspects should be definitely considered in future large retrospective/prospective trials including tumor budding in stage IV CRC with liver metastases.

## Data Availability Statement

The datasets generated for this study are available on request to the corresponding author.

## Ethics Statement

Ethical approval was obtained for the use of all tissue and data in this study (KEK#2017-01803). The studies involving human participants were reviewed and approved by Kantonale Ethikkommission Bern. Written informed consent for participation was not required for this study in accordance with the national legislation and the institutional requirements.

## Author Contributions

AB and CS: writing and editing of the original draft. HD, GB, and IZ: reviewing and editing. AL: writing, reviewing, and editing.

### Conflict of Interest

The authors declare that the research was conducted in the absence of any commercial or financial relationships that could be construed as a potential conflict of interest.
